# Value of acoustic cardiography in the clinical diagnosis of coronary heart disease

**DOI:** 10.1002/clc.23694

**Published:** 2021-09-05

**Authors:** Fu Wei Zhang, Yi Xue Zhang, Liang Yi Si, Mo Shui Chen, Wei Wei Wang, Hai Rong Liang

**Affiliations:** ^1^ Division of Cardiology Third Affiliated Hospital of Chongqing Medical University Chongqing China; ^2^ Division of Cardiology Haikou People's Hospital, Central South University Haikou China

**Keywords:** acoustic cardiography, coronary artery disease, ventricular systolic dysfunction

## Abstract

**Background:**

To investigate the clinical value of acoustic cardiography in the diagnosis of coronary artery disease (CAD) and post‐percutaneous coronary intervention (PCI) early asymptomatic left ventricular systolic dysfunction.

**Methods:**

Inpatients in the department of cardiology were included in the research (*n* = 315); including 180 patients with angina pectoris and 135 patients with acute anterior wall myocardial infarction after emergency PCI did not present with signs and symptoms of heart failure. Color Doppler echocardiography, brain natriuretic peptide, acoustic cardiography examination were performed. The patients were divided into four groups: non‐CAD group (*n* = 60), CAD group (*n* = 120), MIREF group (EF% < 50%, *n* = 75), and MINEF group (EF% ≥ 50%, *n* = 60).

**Results:**

Acoustic cardiography parameters EMATc, systolic dysfunction index, S3 strength and S4 strength in the MIREF group were higher than those in MINEF group (*p* < .05), and the MINEF group was higher than CAD group (*p* < .05). S3 strength (area under the curve [AUC] 0.67, 95% CI 0.585–0.755, *p* < .001) and S4 strength (AUC 0.617, 95% CI 0.536–0.698, *p* = .011) are useful in the diagnosis of CAD. S3 strength (AUC 0.942, 95% CI 0.807–0.978, *p* < .001) was superior to other indicators in the diagnosis of early left ventricular systolic dysfunction after myocardial infarction.

**Conclusion:**

S4 combined with STT standard change can improve the diagnosis of CAD. Acoustic cardiography can be used as a non‐invasive, rapid, effective, and simple method for the diagnosis of asymptomatic left ventricular systolic dysfunction in the early stage after myocardial infarction.

## 1 INTRODUCTION

The standard 12‐lead electrocardiogram (ECG) is the first choice for the diagnosis of myocardial ischemia, but many patients with angina pectoris do not have typical ST‐T changes. Moreover, advanced investigations, such as coronary CT angiography (CTA) and coronary angiography are invasive and expensive making them difficult to apply comprehensively. As a result, diagnosis of myocardial ischemia may be delayed in some cases. Early ventricular remodeling can occur within 24–72 h after acute myocardial infarction. This remodeling may be characterized by left ventricular enlargement, decreased left ventricular ejection fraction (LVEF) and abnormal regional wall activity, which are the main factors determining the development of further cardiac events and long‐term prognosis after acute myocardial infarction (AMI). Asymptomatic left ventricular systolic dysfunction (LVSD) is a major manifestation of early ventricular remodeling, with a prevalence of up to 30%–60% after AMI. Some experts suggest that patients with asymptomatic LVSD after myocardial infarction with an LVEF less than 50% can be diagnosed with ventricular remodeling.^1^, ^2^, ^3^, ^4^ Because patients with asymptomatic LVSD often lack typical clinical features, they can easily be overlooked by doctors, resulting in aggravation of the disease. Although B‐type brain natriuretic peptide (BNP) and echocardiography can be utilized in the diagnosis of LVSD, they carry several disadvantages including increased cost, the need for professional and technical personnel, and difficulty to achieve dynamic monitoring. Identification of simple, novel parameters that are specific and convenient for predicting coronary artery disease (CAD) is needed, so that appropriate treatments can be initiated as early as possible.

As a result, acoustic cardiography has attracted the attention of researchers as rapid, simple and non‐invasive alternative method for the diagnosis of myocardial ischemia and heart failure. Acoustic cardiography has been reported to have clinical value in the diagnosis of heart failure. For example, some studies have shown that cardiac electromechanical activation time (EMAT), left ventricular systolic time (LVST), and S3 are superior to BNP in the diagnosis of LVSD.^5^ In addition, other experimental studies have shown that reduction in EMAT is related to left ventricular systolic function and electromechanical delay, and an increase in EMAT% could be predictive for re‐admission for heart failure.^6^, ^7^ Furthermore, the third and fourth heart sounds (S3 and S4) are less affected by age and diurnal changes, which increases their utility in evaluating cardiac systolic and diastolic function.^8^, ^9^, ^10^ There are few clinical studies on the value of acoustic cardiography in the diagnosis of coronary heart disease. Some studies have shown that the evaluation for S3 or S4 combined with ECG increases the detection rate of myocardial ischemia by 32%.^11^ When conducting exercise treadmill testing, the use of standard STT changes combined with an S4 score >3.6 as the standard for the diagnosis of coronary heart disease resulted in sensitivity and specificity of 68% and 84% respectively.^12^ However, it is not clear whether acoustic cardiography has value in the diagnosis of asymptomatic LVSD in the early stage after myocardial infarction.

Therefore, we conducted a clinical experimental study to explore the clinical value of acoustic cardiography in the diagnosis of myocardial ischemia and early asymptomatic LVSD in patients with AMI after percutaneous coronary intervention.

## 2 METHODS

### 2.1 Study population and study design

Inpatients in the Department of Cardiology of the third affiliated Hospital of Chongqing Medical University and Haikou people's Hospital from March 05, 2019 to September 30, 2020 were included (*n* = 331, these patients were chosen a priori and patient recruitment stopped after the enrollment goals were met,16 patients were excluded due to poor ECG quality). Among them, 180 patients with angina pectoris were examined by acoustic cardiography, cardiac doppler ultrasound, ECG and troponin I on admission in order to exclude myocardial infarction (MI was excluded on the basis of troponin I, ECG and physician diagnosis). After elective coronary angiography (CAG), the patients were divided into two groups: 60 cases without CAD (normal CAG or mild stenosis, non‐CAD) and 120 cases with CAD (CAG indicated at least one major coronary artery with stenosis >50%, CAD). Another 135 patients with asymptomatic acute anterior wall myocardial infarction after emergency PCI were included (all occurring within 24 h, with increased troponin I levels, ST segment elevation: and pathological Q wave on ECG, an angiography showing severe stenosis or occlusion of the anterior descending branch). Color Doppler echocardiography, BNP and acoustic cardiography were performed after 72 h. According to EF%, study subjects were divided into two groups: those with LVSD (EF% < 50% MIREF, 75 cases) and those with normal left ventricular systolic function (EF% ≥ 50%, MINEF, 60 cases).

We collected the subjects' general information including age, gender, heart rate, blood pressure, current medications, medical history, standard 12‐lead ECG. CAD was defined as horizontal or down‐sloping ST segment depression ≥0.5 mm in ≥2 contiguous leads or T wave inversion ≥1 mm in ≥2 contiguous leads. Exclusion criteria were as follows: Pre‐excitation syndrome, atrial fibrillation, atrioventricular block, intraventricular block, chronic obstructive pulmonary disease(COPD), valvular heart disease, congenital cardiovascular disease, pericarditis, myocarditis, cardiomyopathy, severe liver and kidney disease, mechanical ventilation, cardiac pacemaker. Written informed consent was obtained from each patient prior to participation. The study was approved by the local ethics committees of the participating institutions.

### 2.2 Acoustic cardiography

**FIGURE 1:**
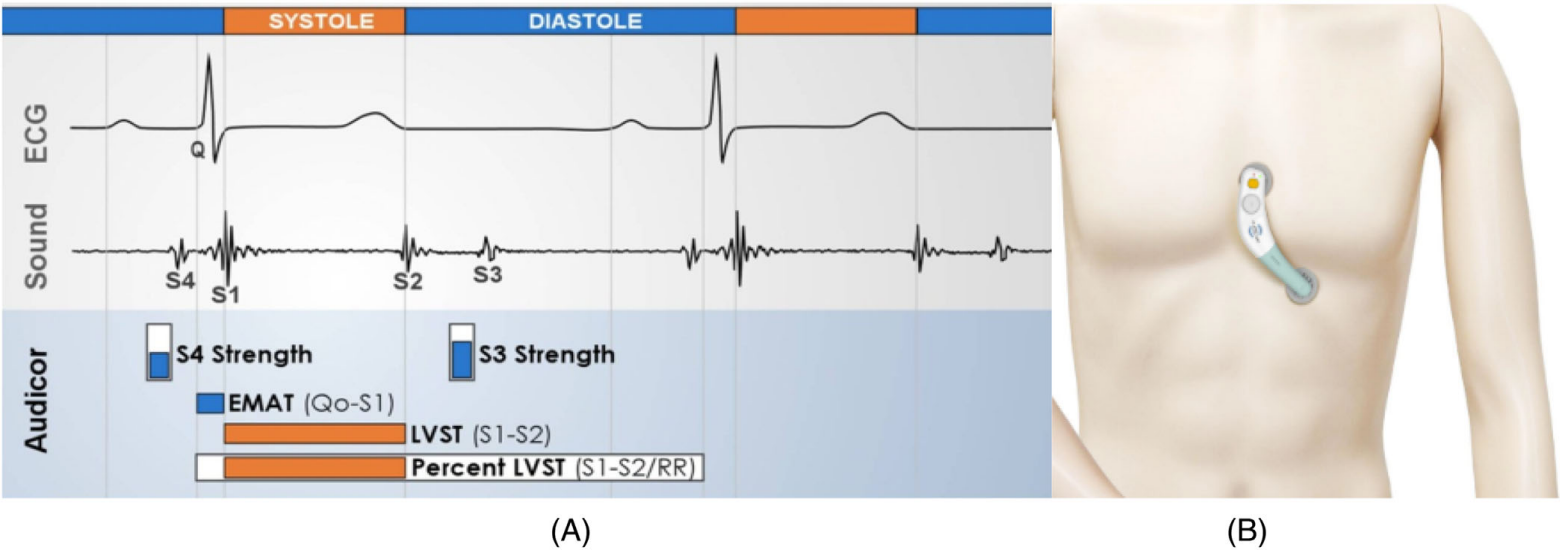
Acoustic cardiography parameters (A) and usage mode (B). EMAT, electromechanical activation time; LVST, left ventricular systolic time; S3, third heart sound; S4, fourth heart sound; SDI, systolic dysfunction index

Each subject underwent acoustic cardiography examination in a supine position (Figure 1(B)). Acoustic cardiography is a technique that Simultaneous ECG and heart sound data from the V3/V4 standard precordial position were analyzed by the computerized algorithm to calculate the EMAT, S3, S4, systolic dysfunction index (SDI).^13^ This algorithm was previously validated by blinded expert interpretation of heart sound tracings. At least three sequential recordings were performed on each study subject and the average value of each variable were used for analysis.

The following acoustic cardiography parameters were evaluated in this study:

1. EMAT: the time from the Q wave onset to the mitral component of the first heart sound (S1). EMATc indicates the proportion of the cardiac cycle occupied by EMAT (Figure 1(A)).
2. Fourth heart sound (S4) strength: the measurement of the intensity and consistency of the S4; one value between 0 and 10 is reported (Figure 1(A)).
3. Third heart sound (S3) strength: the measurement of the intensity and consistency of the S3; one value between 0 and 10 is reported (Figure 1(A)).
4. SDI: The SDI value is a mathematical combination of QRS duration, QR interval, EMAT, and the RR interval; it undergoes a nonlinear transformation and is mapped onto a scale of 0–10.

### 2.3 Echocardiography

Echocardiography was performed according to American Society of Echocardiography guidelines (equipment: Vivid 7, Vingmed‐General Electric, IE33, Phillips, Andover). We used the biplane Simpson's method to calculate end‐diastolic and end‐systolic volumes, and these volumes were used to calculate LVEF.

### 2.4 Statistical analysis

Statistical analyses were performed using SPSS, version 20 (SPSS, Inc., Chicago, IL). A two‐sided *p* value <.05 was considered statistically significant. Data were described as means and standard deviations for continuous variables and frequency and proportions for categorical variables. Comparisons between groups were tested using student's *t* test for normally distributed data and Mann–Whitney *U* test for skewed data. Receiver operating characteristic (ROC) curves were generated to determine area under the curve (AUC), sensitivity, specificity, positive likelihood ratio (LR+), and negative likelihood ratio (LR−) for predicting ischemia and AMI with early asymptomatic LVSD (EALVSD). Binary logistic regression analysis was used to identify independent risk factors for AMI with EALVSD.

## 3 RESULTS

### 3.1 Characteristics of study subjects

Basic demographics, medication use and clinical characteristics of study subjects are summarized in Table 1.

**TABLE 1:** Demographics and clinical characteristics

Variables	Non‐CAD	CAD	MINEF	MIREF	*p*
	*N* = 60	*N* = 120	*N* = 60	*N* = 75	
Male, *n* (%)	36 (60%)	71 (59%)	31 (52%)	41 (55%)	.677
Diabetes, *n* (%)	14 (25%)	37 (31%)	20 (33%)	28 (37%)	.368
ACEI/ARB, *n* (%)	19 (32%)	53 (44%)	37 (62%)^a^ ^,^ ^b^	46 (61%)^a^ ^,^ ^b^	.001
Betablockers, *n* (%)	15 (25%)	52 (43%)	31 (52%)	35 (47%)	.163
CCB, *n* (%)	20 (33%)	42 (35%)	15 (25%)	25 (33%)	.563
Hypertension, *n* (%)	27 (45%)	61 (51%)	33 (55%)	38 (51%)	.749
Nitrates, *n* (%)	25 (42%)	81 (68%)	47 (78%)^a^ ^,^ ^b^	61 (81%)^a^ ^,^ ^b^	<.001
Diuretics, *n* (%)	10 (17%)	25 (21%)	13 (22%)	21 (28%)	.445
Age, years	63.5 ± 7.5	62.2 ± 9.3	61.7 ± 8.9	64.7 ± 8.9	.177
Heart rate (bpm)	75.8 ± 9.0	75.2 ± 9.2	75.4 ± 10.7	88.6 ± 9.9^a^ ^,^ ^b^ ^,^ ^c^	<.001
SBP (mmHg)	139.5 ± 18.89	137.8 ± 18.8	135.2 ± 21.5	142.8 ± 16.4	.118
DBP (mmHg)	77.0 ± 11.6	77.1 ± 8.9	75.5 ± 9.5	78.3 ± 7.3	.369
S3	3.13 ± 0.86	3.66 ± 0.95^a^	4.07 ± 0.73^a^ ^,^ ^b^	5.86 ± 0.97^a^ ^,^ ^b^ ^,^ ^c^	<.001
S4	3.67 ± 0.72	4.01 ± 0.96	4.27 ± 1.11^a^ ^,^ ^b^	5.77 ± 1.07^a^ ^,^ ^b^ ^,^ ^c^	.001
EMATc	10.45 ± 2.19	10.82 ± 2.12	11.71 ± 2.10^a^ ^,^ ^b^	15.05 ± 2.15^a^ ^,^ ^b^ ^,^ ^c^	.001
SDI	3.19 ± 0.68	3.41 ± 0.95	3.86 ± 0.89^a^ ^,^ ^b^	5.79 ± 1.18^a^ ^,^ ^b^ ^,^ ^c^	.001
LVEF%	56.52 ± 4.82	56.03 ± 3.48	55.48 ± 3.96	43.28 ± 4.01^a^ ^,^ ^b^ ^,^ ^c^	<.001
BNP (pg/ml)			101.54 ± 49.15	385.92 ± 228.39^c^	<.001

Abbreviations: ACEI/ARB, angiotensin converting enzyme inhibitor/angiotensin II receptor blocker; BNP, B‐type brain natriuretic peptide; CAD, coronary artery disease; CCB, calcium channel blocker; DBP, diastolic blood pressure; EMATc, electromechanical activation time divided by the cardiac cycle length; MINEF, acute anterior myocardial infarction with normal ejection fraction; MIREF, acute anterior myocardial infarction with reduced ejection fraction; non‐CAD, normal coronary angiography or mild stenosis; S3, third heart sound; S4, fourth heart sound; SBP, systolic blood pressure; SDI, systolic dysfunction index.

^a^ *p* < .05 for the comparison with patients in the non‐CAD.

^b^ *p* < .05 for the comparison with patients in the CAD group.

^c^ *p* < .05 in comparison with patients in the MINEF group.

### 3.2 Diagnostic characteristics of acoustic cardiography for detecting coronary artery disease

**FIGURE 2:**
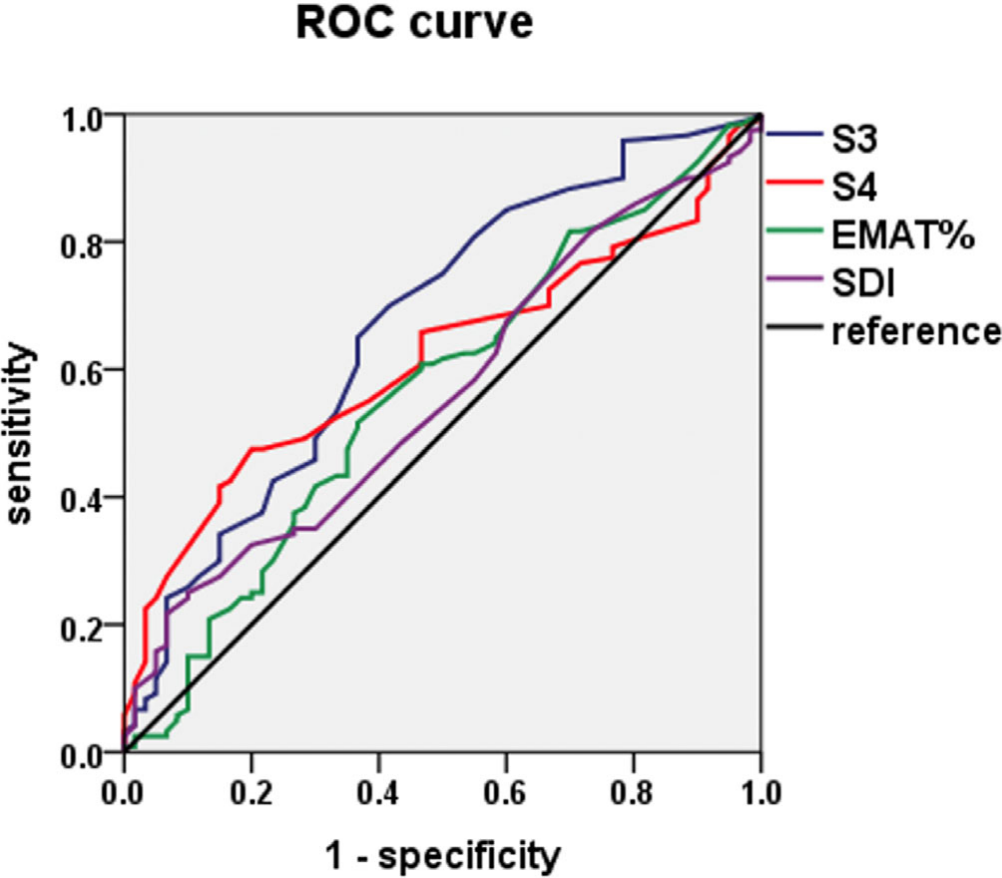
Receiver operating characteristic curves of S3, S4, EMATc, and SDI for detecting coronary artery disease. The blue purple is for S3, and the AUC is 0.670 (95% CI 0.585–0.755, *p* = .000). The red curve is for S4 and the AUC is 0.617 (95% CI 0.536–0.698, *p* = .011). The green curve is for EMATc and the AUC is 0.567 (95% CI 0.477–0.656, *p* = .146). The purple curve is for SDI and the AUC is 0.562 (95% CI 0.476–0.649, *p* = .174). EMATc, electromechanical activation time divided by the cardiac cycle length; S3, third heart sound; S4, fourth heart sound; SDI, systolic dysfunction index

ROC curves analysis was used to determine the value of various acoustic cardiographic parameters for predicting CAD. As shown in Figure 2 and Table 2, the area under the ROC curve (AUC) for S3 strength was 0.67 (95% confidence interval [CI] 0.702–0.896, *p* = .000). With an optimal cutoff point of 3.05, S3 strength produced a sensitivity of 70% and a specificity of 58%. The AUC for S4 was 0.617 (95% CI 0.536–0.698, *p* = .011). With an optimal cutoff point of 4.25, S4 strength produced a sensitivity of 48% and a specificity of 80%. The sensitivity and specificity to detect ischemia was 69% and 80%, respectively, when S4 strength was added to ST‐T wave criteria.

**TABLE 2:** Performance of acoustic cardiographic parameters and STT change to detect coronary artery disease

Parameter	Threshold	Sensitivity	Specificity	LR+	LR−
S3	≥3.05	84/120 (70%)	35/60 (58%)	1.67	0.52
S4	≥4.25	58/120 (48%)	48/60 (80%)	2.4	0.65
S3 ≥ 3.05 or S4 ≥ 4.25		103/120 (86%)	30/60 (50%)	1.72	0.28
STT change		46/120 (38%)	54/60 (90%)	3.8	0.69
S4 ≥ 4.25 or STT change		83/120 (69%)	48/60 (80%)	3.45	0.39
S3 ≥ 3.05 or STT change		93/120 (77%)	33/60 (55%)	2.07	0.13

*Note*: STT change: horizontal or down‐sloping ST segment depression ≥0.5 mm in ≥2 contiguous leads or T wave inversion ≥1 mm in ≥2 contiguous leads.

Abbreviations: LR+, positive likelihood ratio; LR−, negative likelihood ratio; S3, third heart sound; S4, fourth heart sound.

### 3.3 Diagnostic characteristics of acoustic cardiography for detecting AMI with early asymptomatic left ventricular systolic dysfunction

**FIGURE 3:**
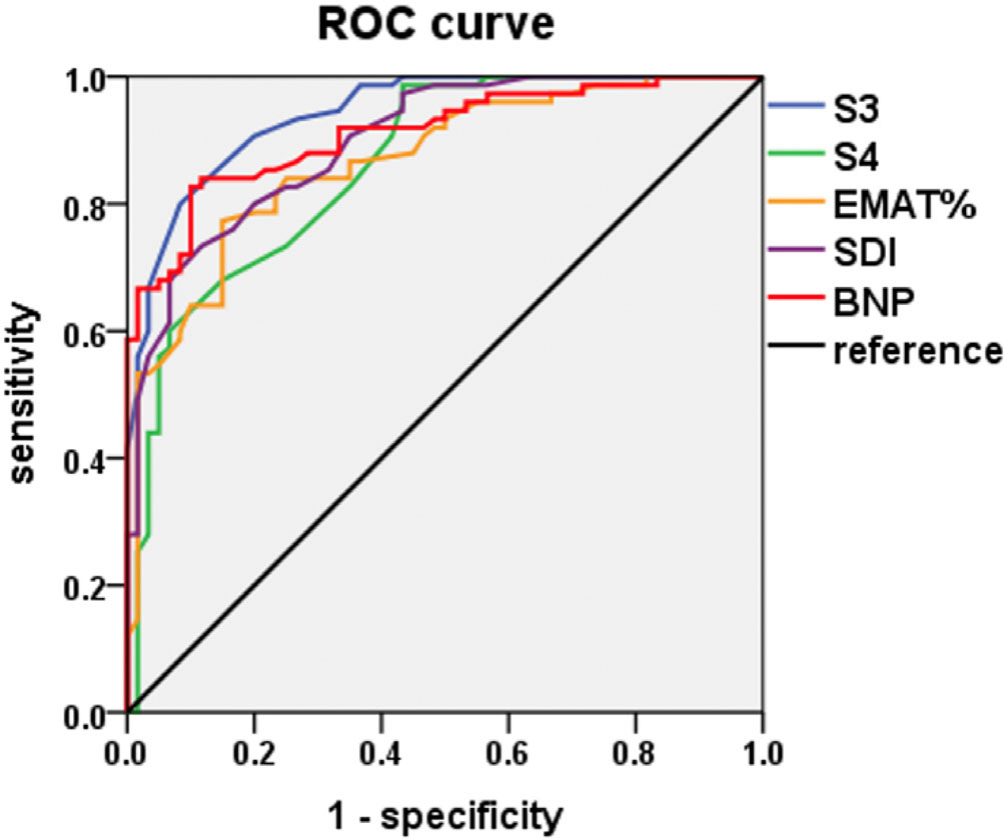
Receiver operating characteristic curves of S3 strength, EMATc, SDI, and BNP for differentiating MIREF from MINEF. The green curve represents S4 strength, with an AUC of 0.862 (95% CI 0.799–0.924, *p* < .001). The blue curve represents S3 strength, with an AUC of 0.942 (95% CI 0.807–0.978, *p* < .001). The red curve represents BNP, with an AUC of 0.911(95% CI 0.864–0.959, *p* < .001). The yellow curve represents EMATc, with an AUC of 0.867 (95% CI 0.807–0.927, *p* < .001). The purple curve represents SDI, with an AUC of 0.899 (95% CI 0.849–0.949, *p* < .001). BNP, B‐type brain natriuretic peptide; EMATc, electromechanical activation time divided by the cardiac cycle length; S3, third heart sound; S4, fourth heart sound; SDI, systolic dysfunction index

Figure 3 displays the ROC curve analyses of various acoustic cardiography parameters and BNP for predicting AMI with early EALVSD. Figure 3 and Table 3 we examined the AUC, cutoff values, specificity, sensitivity and likelihood ratios for each diagnostic parameter. S3 strength was the best predictor to detect AMI with EALVSD, and S3 strength >4.85 yielded an AUC of 0.942 with 80% sensitivity and 92% specificity. Similarly, BNP >139.3 pg/ml yielded an AUC of 0.911 with 82% sensitivity and 90% specificity. The sensitivity and specificity to detect AMI with EALVSD were 93% and 83% respectively, when S3 was added to BNP.

**TABLE 3:** Performance of acoustic cardiographic parameters and BNP to detect MINEF and MIREF

Parameter	Cutoff value	Sensitivity	Specificity	LR+	LR−
S3	≥4.85	60/75 (80%)	5/60 (92%)	10	0.22
EMATc	≥13.6%	58/75 (77%)	9/60 (85%)	5.13	0.15
SDI	≥4.85	55/75 (73%)	8/60 (87%)	5.6	0.31
BNP (pg/ml)	≥139.3	62/75 (82%)	6/60 (90%)	8.20	0.20
BNP (pg/ml)	≥100	65/75 (87%)	20/60 (67%)	2.64	0.19
S3 ≥ 4.85 or BNP ≥ 139.3		70/75 (93%)	10/60 (83%)	5.47	0.08

Abbreviations: BNP, B‐type brain natriuretic peptide; EMATc, electromechanical activation time divided by the cardiac cycle length; LR+, positive likelihood ratio; LR−, negative likelihood ratio; S3, third heart sound; SDI, systolic dysfunction index.

## 4 DISCUSSION

Our experimental evidence in this study indicates that acoustic cardiography may have clinical value in the diagnosis of coronary heart disease. The sensitivity of S3 Strength, S4 Strength and ST‐T change for the diagnosis of CAD respectively is 70%, 48%, and 38%, respectively, with specificity of 58%, 80%, and 90% respectively. The sensitivity and specificity of S3 Strength combined with ST‐T change is 69% and 80%, respectively. Acoustic cardiography combined ECG could improve the clinical diagnosis rate of coronary heart disease in patients with angina. Many other researchers have studied the diagnostic value of acoustic cardiography in coronary heart disease. One study found that in 40% of patients with myocardial ischemia or reperfusion model during PCI developed an S3 and S4 earlier than ST segment changes. After myocardial ischemia reperfusion, the duration of S4 and S3 were longer.^14^ Acoustic cardiography can be used to identify intravascular murmurs caused by coronary artery stenosis and detect mechanical dysfunction induced by acute myocardial ischemia.^15^, ^16^ In general, our study indicates that S4 strength combined with ST‐T changes of the ECG can improve the diagnosis of coronary heart disease. We did not use fractional flow reserve (FFR) as a diagnostic criterion for myocardial ischemia. It is the limitation of our experiment. The reason that we did not evaluate myocardial ischemia using FFR was that FFR is usually performed in our hospital when the coronary artery is about 75%. When it is significantly less than or more than 75%, generally, it will not be considered, which may be out of consideration for operation time, patients' safety, drug safety, and so on.

In clinical practice, AMI often causes heart failure, in particular within the week following the acute event. Although emergency PCI can reduce the incidence of arrhythmia, cardiogenic shock and heart failure following AMI, there are still some patients who suffer from heart failure. When signs and clinical symptoms are obvious, doctors can diagnose and treat efficaciously. However, some patients with early LVSD and atypical clinical symptoms may be missed by the doctor. Moreover, it is difficult to monitor heart failure in real time using color Doppler ultrasound and BNP. The results of this study suggest that S3 strength is superior to other parameters in the diagnosis of early LVSD after myocardial infarction. The sensitivity and specificity of S3, EMAT%, SDI, BNP refers to the results section for the detailed numbers. The specificity of acoustic cardiography to detect LVSD in this study was higher than that of BNP, and the combination of S3 and BNP would further improve the sensitivity. Therefore, in the early diagnosis of asymptomatic LVSD after PCI, acoustic cardiography was superior to BNP. Moreover, it was easier to acquire results and could be monitored dynamically at any time. In conclusion, acoustic cardiography is a fast, effective, and simple method for monitoring cardiac function after myocardial infarction.

There are several clinical studies that support the value of acoustic cardiography in the diagnosis of heart failure; some studies suggest that SDI >5 correlated with a LVEF <50%, whereas SDI >7.5 correlates with EF <35% and elevated left ventricular filling pressure.^17^ The performance of acoustic cardiography to diagnose acute heart failure (sensitivity and specificity of S3 ≥ 5.0 + %EMAT ≥ 14.4% were 69% and 100%, respectively) was better than BNP in the “gray zone” of 100 pg/ml < BNP < 500 pg/ml.^18^ Additional studies show that SDI is more sensitive and specific in the diagnosis of heart failure with LVEF ≤35%, while S3 has high sensitivity and specificity in the diagnosis of diastolic heart failure.^19^

In the follow‐up observation of patients with chronic heart failure, the patients with SDI ≥5 or S3 strength ≥4 had increased mortality. Acoustic cardiography can predict the prognosis of patients with chronic heart failure, and be used for adjustment of the patient's treatment plan based on the results of EMAT to significantly reduce adverse events. The monitoring of night time EMAT is a method to predict adverse events of acute heart failure with results better than hemodynamic monitoring.^20^, ^21^, ^22^, ^23^ Other studies show that monitoring with acoustic cardiography can be used as a fast, simple, and effective method to predict early systolic dysfunction and to screen patients with sleep apnea syndrome.^24^, ^25^, ^26^

In conclusion, S4 strength combined with ST‐T changes of the ECG can improve the diagnosis of coronary heart disease; Acoustic cardiography can be used as a fast, simple, inexpensive and effective method to diagnose asymptomatic LVSD in the early weeks after AMI.

## 5 LIMITATIONS OF THE STUDY

The sample size of this study was small; larger studies are needed to confirm these findings. Only the ejection fraction was recorded during echocardiographic examination which did not allow exploration of other cardiac parameters. We did not use FFR as a diagnostic criterion for myocardial ischemia. It is the limitation of our experiment, in subsequent experiment, we will improve the evaluation and diagnostic criteria of myocardial ischemia.

## CONFLICT OF INTEREST

The authors declare that they have no conflicts of interest.

## Data Availability

All data generated or analysed during this study are included in this published article .The datasets used and/or analysed during the current study are available from the corresponding author on reasonable request.

## References

[1] Cohn JN , Ferrari R , Sharpe N . Cardiac remodeling ‐ concepts and clinical implications: a consensus paper from an international forum on cardiac remodeling. Behalf of an international forum on cardiac remodeling. J Am Coll Cardiol. 2000;35(3):569‐582. 10.1016/s0735-1097(99)00630-0 10716457

[2] Yalta K , Yilmaz MB , Yalta T , et al. Late versus early myocardial remodeling after acute myocardial infarction: a comparative review on mechanistic insights and clinical implications. J Cardiovasc Pharmacol Ther. 2020;25(1):15‐26. 10.1177/1074248419869618 31416353

[3] Weir RAP , McMurray JJV , Velazquez EJ . Epidemiology of heart failure and left ventricular systolic dysfunction after acute myocardial infarction: prevalence, clinical characteristics, and prognostic importance. Am J Cardiol. 2006;97(10A):13F–25F. 10.1016/j.amjcard.2006.03.005 16698331

[4] Echouffo‐Tcheugui JB , Erqou S , Butler J , Yancy CW , Fonarow GC . Assessing the risk of progression from asymptomatic left ventricular dysfunction to overt heart failure: a systematic overview and meta‐analysis. JACC Heart Fail. 2016;4(4):237‐248. 10.1016/j.jchf.2015.09.015 26682794

[5] Kosmicki DL , Collins SP , Kontos MC , et al. Noninvasive prediction of left ventricular systolic dysfunction in patients with clinically suspected heart failure using acoustic cardiography. Congest Heart Fail. 2010;16(6):249‐253. 10.1111/j.1751-7133.2010.00191.x 21091608

[6] Efstratiadis S , Michaels AD . Computerized acoustic cardiographic electromechanical activation time correlates with invasive and echocardiographic parameters of left ventricular contractility. J Card Fail. 2008;14(7):577‐582. 10.1016/j.cardfail.2008.03.011 18722323

[7] Chao TF , Sung SH , Cheng HM , et al. Electromechanical activation time in the prediction of discharge outcomes in patients hospitalized with acute heart failure syndrome. Intern Med. 2010;49(19):2031‐2037. 10.2169/internalmedicine.49.3944 20930426

[8] Dillier R , Zuber M , Arand P , Erne S , Erne P . Assessment of systolic and diastolic function in heart failure using ambulatory monitoring with acoustic cardiography. Ann Med. 2011;43(5):403‐411. 10.3109/07853890.2010.550309 21361859

[9] Dillier R , Zuber M , Arand P , Erne S , Erne P . Assessment of systolic and diastolic function in asymptomatic subjects using ambulatory monitoring with acoustic cardiography. Clin Cardiol. 2011;34(6):384‐388. 10.1002/clc.20891 21538386 PMC6652310

[10] Dillier R , Kobza R , Erne S , et al. Noninvasive detection of left‐ventricular systolic dysfunction by acoustic cardiography in atrial fibrillation. Cardiol Res Pract. 2010;2011:173102. 10.4061/2011/173102 20981304 PMC2958491

[11] Lee E , Drew BJ , Selvester RH , Michaels AD . Diastolic heart sounds as an adjunctive diagnostic tool with ST criteria for acute myocardial ischemia. Acute Card Care. 2009;11(4):229‐235. 10.1080/17482940903203071 19995262

[12] Zuber M , Erne P . Acoustic cardiography to improve detection of coronary artery disease with stress testing. World J Cardiol. 2010;2(5):118‐124. 10.4330/wjc.v2.i5.118 21160713 PMC2998883

[13] Wen YN , Lee AP , Fang F , et al. Beyond auscultation: acoustic cardiography in clinical practice. Int J Cardiol. 2014;172(3):548‐560. 10.1016/j.ijcard.2013.12.298 24529949

[14] Lee E , Drew BJ , Selvester RH , Michaels AD . Sequence of electrocardiographic and acoustic cardiographic changes and angina during coronary occlusion and reperfusion in patients undergoing percutaneous coronary intervention. Ann Noninvasive Electrocardiol. 2009;14(2):137‐146. 10.1111/j.1542-474X.2009.00288.x 19419398 PMC6931998

[15] Azimpour F , Caldwell E , Tawfik P , et al. Audible coronary artery stenosis. Am J Med. 2016;129(5):515‐521.e3. 10.1016/j.amjmed.2016.01.015 26841299

[16] Luciani M , Saccocci M , Kuwata S , et al. Reintroducing heart sounds for early eetection of acute myocardial ischemia in a porcine model ‐ correlation of acoustic cardiography with gold standard of pressure‐volume analysis. Front Physiol. 2019;10:1090. 10.3389/fphys.2019.01090 31507452 PMC6713932

[17] Wang S , Lam YY , Liu M , et al. Acoustic cardiography helps to identify heart failure and its phenotypes. Int J Cardiol. 2013;167(3):681‐686. 10.1016/j.ijcard.2012.03.067 22456263

[18] Zuber M , Kipfer P , Attenhofer Jost CH . Usefulness of acoustic cardiography to resolve ambiguous values of B‐type natriuretic peptide levels in patients with suspected heart failure. Am J Cardiol. 2007;100(5):866‐869. 10.1016/j.amjcard.2007.04.019 17719335

[19] Wang S , Fang F , Liu M , et al. Rapid bedside identification of high‐risk population in heart failure with reduced ejection fraction by acoustic cardiography. Int J Cardiol. 2013;168(3):1881‐1886. 10.1016/j.ijcard.2012.12.064 23352488

[20] Wang S , Liu M , Fang F , et al. Prognostic value of acoustic cardiography in patients with chronic heart failure. Int J Cardiol. 2016;219:121‐126. 10.1016/j.ijcard.2016.06.004 27323336

[21] Sung SH , Huang CJ , Cheng HM , et al. Effect of acoustic cardiography‐guided management on 1‐year outcomes in patients with acute heart failure. J Card Fail. 2020; Feb;26(2):142‐150. 10.1016/j.cardfail.2019.09.012 31568829

[22] Zhang J , Liu WX , Lyu SZ . Predictive value of electromechanical activation time for in‐hospital major cardiac adverse events in heart failure patients. Cardiovasc Ther. 2020;2020:4532596. 10.1155/2020/4532596 31969933 PMC6961597

[23] Chang CC , Sung SH , Yu WC , et al. Night‐time electromechanical activation time, pulsatile hemodynamics, and discharge outcomes in patients with acute heart failure. ESC Heart Fail. 2015;2(3):184‐193. 10.1002/ehf2.12044 28834674 PMC6410547

[24] Toggweiler S , Odermatt Y , Brauchlin A , et al. The clinical value of echocardiography and acoustic cardiography to monitor patients undergoing anthracycline chemotherapy. Clin Cardiol. 2013;36(4):201‐206. 10.1002/clc.22074 23161530 PMC6649372

[25] Tsai HJ , Tsai YC , Huang JC , et al. Investigation of acoustic cardiographic parameters before and after hemodialysis. Dis Markers. 2019;2019:5270159. 10.1155/2019/5270159 31781303 PMC6874870

[26] Bauer P , Arand P , Radovanovic D , et al. Assessment of cardiac function and prevalence of sleep disordered breathing using ambulatory monitoring with acoustic cardiography – initial results from SWICOS. J Hypertens Cardiol. 2017;2(3):32‐46. 10.14302/issn.2329-9487.jhc-18-1932

